# Deformable versus rigid registration of PET/CT images for radiation treatment planning of head and neck and lung cancer patients: a retrospective dosimetric comparison

**DOI:** 10.1186/1748-717X-9-50

**Published:** 2014-02-10

**Authors:** Dominique Fortin, Parminder S Basran, Tanya Berrang, David Peterson, Elaine S Wai

**Affiliations:** 1 Department of Medical Physics, BC Cancer Agency–Vancouver Island Centre, 2410 Lee Avenue, V8R 6V5 Victoria, British Columbia, Canada; 2 Department of Physics and Astronomy, University of Victoria, Victoria, British Columbia, Canada; 3 Department of Radiation Oncology, BC Cancer Agency–Vancouver Island Center, Victoria, British Columbia, Canada; 4 Department of Surgery, University of British Columbia, Vancouver, British Columbia, Canada

**Keywords:** Deformable, Registration, PET/CT, Treatment, Planning

## Abstract

**Background:**

The purpose of this study is to evaluate the clinical impact of using deformable registration in tumor volume definition between separately acquired PET/CT and planning CT images.

**Methods:**

Ten lung and 10 head and neck cancer patients were retrospectively selected. PET/CT images were registered with planning CT scans using commercially available software. Radiation oncologists defined two sets of gross tumor volumes based on either rigidly or deformably registered PET/CT images, and properties of these volumes were then compared.

**Results:**

The average displacement between rigid and deformable gross tumor volumes was 1.8 mm (0.7 mm) with a standard deviation of 1.0 mm (0.6 mm) for the head and neck (lung) cancer subjects. The Dice similarity coefficients ranged from 0.76-0.92 and 0.76-0.97 for the head and neck and lung subjects, respectively, indicating conformity. All gross tumor volumes received at least 95% of the prescribed dose to 99% of their volume. Differences in the mean radiation dose delivered to the gross tumor volumes were at most 2%. Differences in the fraction of the tumor volumes receiving 100% of the radiation dose were at most 5%.

**Conclusions:**

The study revealed limitations in the commercial software used to perform deformable registration. Unless significant anatomical differences between PET/CT and planning CT images are present, deformable registration was shown to be of marginal value when delineating gross tumor volumes.

## Background

Advances in imaging have made a profound impact in the diagnosis and management of cancer. Techniques such as Positron Emission Tomography (PET) and Computed Tomography (CT) are now widely used for staging and tumor delineation for lung and head and neck cancers [1-6]. In particular, accurately defining target volumes in radiation treatment planning is crucial to ensure proper coverage of the tumors and spare organs at risk.

While PET images provide details on the extent and intensity of the metabolically active tumor, CT images define the anatomical details of the tumor and surrounding healthy tissues. To better correlate the location of the ^18^F-fluorodeoxyglucose (FDG) avid tumors from the combined PET/CT images with the planning CT scan, software can be deployed to co-register the images. Rigid image registration (RIR) of the two CT images can effectively align the PET to the planning CT images to accurately define the volumes for radiation treatment [7].

Many cancer centers have access to a diagnostic PET/CT scanner, but not necessarily to a PET/CT radiation therapy simulator [8]. Thus, PET/CT images are often acquired at different time-points and locations in the radiation treatment planning process, using various patient accessories and imaging equipment. Unless a dedicated PET/CT scanner is used for radiation treatment simulation, the patient anatomy on the PET/CT does not always correspond to that of the planning CT. This can be further complicated by changes in weight in the patient between scans, changes in the positioning of the patient, and soft tissue displacements due to breathing, peristaltic, cardiac or involuntary motion. Sophisticated registration methods have become available in the clinic to account for these motions [9]. Deformable image registration (DIR) attempts to correct for these effects by providing a mapping between volume elements in one image to the corresponding volume elements in a second image.

Although many studies investigating the performance and utility of DIR have been conducted [10-16], no dosimetric information regarding the clinical impact of DIR of PET/CT to planning CT has been found in the literature, nor any clinical studies validating the software used. The purpose of this retrospective study is to assess the utility and efficacy of DIR between PET/CT and planning CT images for radiation therapy patients. The impact of DIR on tumor volume definition is investigated using radiation treatment plans for patients with lung or head and neck cancer by quantitatively comparing tumor volumes defined with RIR and DIR PET/CT images using metrics such as the Dice similarity coefficient, displacement of the center of mass, and radiation dose received.

## Methods and materials

Research ethics was obtained from the University of British Columbia research ethics board in conjunction with the BC Cancer Agency. Twenty cancer patients between the age of 46 and 74 years were retrospectively selected from the treatment-planning database: 10 lung and 10 head and neck patients. All patients underwent curative cancer treatment at the BC Cancer Agency in Victoria between May 2012 and February 2013 using Intensity modulated radiotherapy (IMRT).

The CT simulation for head and neck patients was performed at the treatment site with a GE Optima CT580 scanner and patients were immobilized with a thermoplastic shell. Patients were then sent to an imaging center with their shell, and imaged using a GE Discovery 600/690 PET/CT scanner. Full body scans were acquired with the patients immobilized in their thermoplastic shell on a flat-top couch, and a high-resolution PET/CT of the head and neck region was generated for radiation treatment purposes. PET/CT scans were performed 1-15 days after the acquisition of the planning CT scans. Lung patients were immobilized on a flat-top couch using an in-house t-bar with their arms above their heads for the acquisition of planning CT scans. A whole body PET/CT scan was acquired 1-10 weeks prior to the planning CT scan as part of the routine diagnostic protocol for lung cancer. Patients were positioned on a conventional diagnostic curved couch without the t-bar, with a pillow for head support, and arms typically down. No respiratory gating techniques were used during the acquisition of the planning CT and PET/CT scans as the tumor volumes were located in the superior lobes of the lungs and the mediastinum, where motion due to breathing is less pronounced than regions near the diaphragm. Digital images of the PET/CT and planning CT scans were made accessible via network connections in DICOM format, and imported into a treatment-planning database.

The PET/CT images were fused with the planning CT scans within the treatment planning system (Eclipse, Varian Medical Systems, Palo Alto CA). Rigid registration of the PET/CT and planning CT images was performed using the Varian Rigid Registration package (version 10.0). The PET image intensities were displayed in units of Standardized Uptake Value (SUV) based on the patient’s body weight, recorded during the PET/CT acquisition and available in the DICOM data. Settings for the PET image display were adjusted such that the minimum SUV intensity was 2.0 Bq/ml.

Routine treatment planning was performed for all subjects prior to this. Normal tissue and tumor volume contouring was done by radiation oncology and dosimetry staff within the planning system and a treatment plan was generated. The standard treatment prescription dose for patients with head and neck cancers was 70 Gy in 35 fractions, delivered with intensity modulated radiotherapy, and for patients with lung cancers was 40-60 Gy in 2.0-2.5 Gy fractions, delivered using a three-dimensional conformal radiation therapy treatment plan.

For this study, automatic deformable registration (DIR) of the PET/CT images with the planning CT scans was performed on a GE Advantage Workstation 4.3 (GE Integrated Registration, GE Medical Systems, Cleveland OH). As the registration software is proprietary, there was limited control of its actions and the algorithms underpinnings. The performance of the registration was validated using a cylindrical CT image quality phantom: known deformations were applied to the images of the phantom, and the deformable registration software was then used in an attempt to obtain the original image back [17]. The fidelity of image quality was then quantitatively analyzed. This study showed that various deformations as large as 2-3 cm were recovered using the deformable registration software.

**Figure 1:**
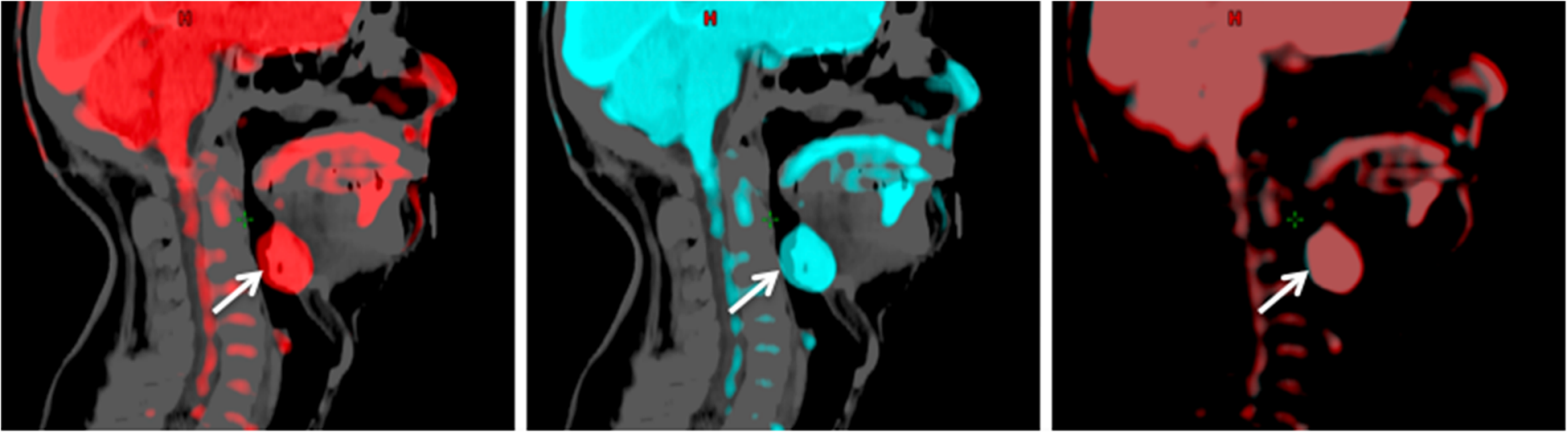
**Sagittal view of the fused PET and planning CT scans for a head and neck cancer subject.** Results from the RIR (left) and DIR (center), and fused view of the RIR and DIR PET (right) are shown. The difference in the position of the GTV for the primary tumor, indicated by a white arrow, is measured to be 1.1 mm between RIR and DIR.

The algorithm was observed to register the CT images from the PET/CT scans with the planning CT scan in two steps: a RIR followed by a DIR. During this process, each PET voxel was mapped to a new position based on the transformations used in the CT-CT registration, resulting in a new PET/CT dataset that was deformably registered with the planning CT. To improve the quality of the registration and in some cases prevent the DIR algorithm from failing, CT images from the combined PET/CT scans outside the volume range of the planning CT scans were manually removed from the series. The resulting rigid and deformable PET image registration against the planning CT scan is shown in Figure 1 in the case of one head and neck cancer subject.

**Figure 2:**
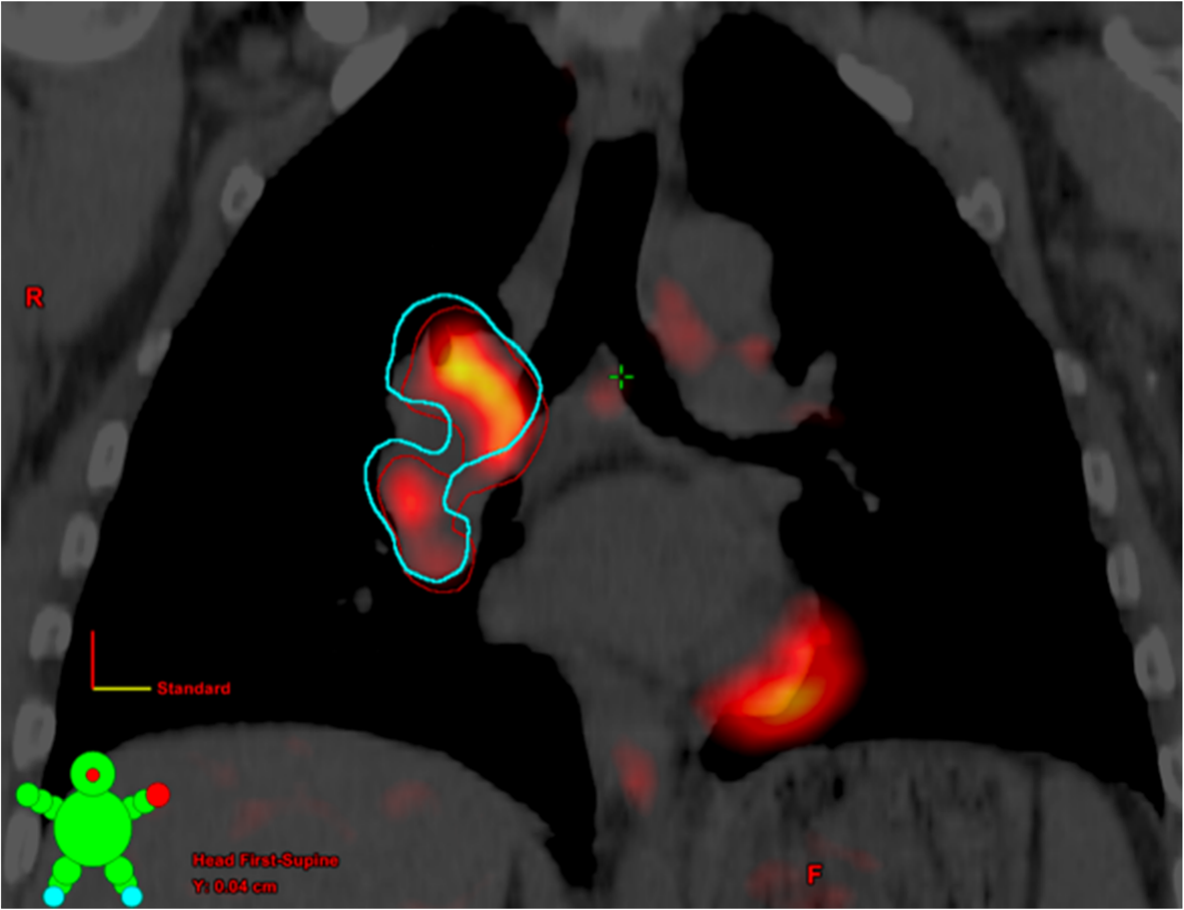
**Coronal view of the planning CT with fused PET for a lung cancer subject.** The GTV contours generated using the RIR and DIR PET images are shown in red and blue, respectively. An intensity threshold equivalent to SUV ≥ 2.0 was used for the PET image.

The registered images were then imported back into the radiation treatment planning system. Five radiation oncologists were asked to manually contour 2 sets of GTVs on the planning CT, one aided by the RIR PET image series and the second aided by the DIR PET image series as shown in Figure 2. Diagnostic radiology reports were made available to assist the radiation oncologists in their contouring, but information regarding the type of registration used was removed. The position and volume of the GTVs and radiation doses delivered to each GTV using the original treatment plan developed for each subject prior to the study were compared using available tools within the planning system. In the case of subjects with multiple tumor foci, individual GTVs were contoured for the largest 2 cancer sites only.

The volumetric analysis of the difference between GTVs obtained with RIR and DIR consisted in determining the Dice similarity coefficient, DSC = 2 [V_RIR_ ∩ V_DIR_]/[V_RIR_ + V_DIR_]. This metric has values ranging from 0 for no overlap to 1 for perfect agreement between volumes.

## Results

### Head and neck cancer analysis

**Figure 3:**
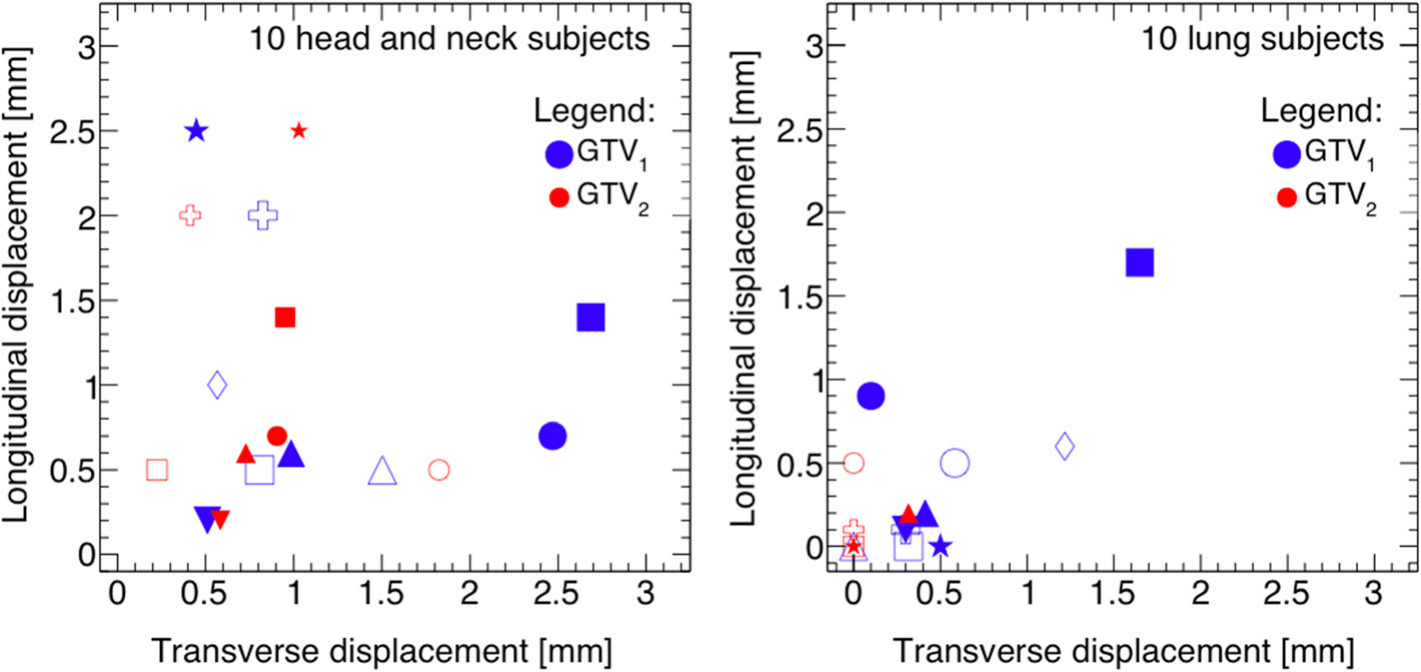
**Comparing the position of GTVs defined using RIR and DIR PET images.** The displacement along the z-axis is shown as a function of the displacement in the transverse plane for the head and neck (left) and lung (right) cancer subjects. One point falls beyond the range of the histogram for a head and neck subject, where displacements of 4.5 mm in the transverse plane and 0.5 mm along the longitudinal axis are observed. Ten different symbols are used for the 10 different subjects. The primary tumors are indicated by larger symbols in blue, whereas secondary nodes are drawn using smaller matching symbol in red.

Ten head and neck cancer patients were selected for this study. Primary sites varied greatly and ranged from the larynx to the nasal cavity, and for eight out of ten patients, multiple nodes were observed. The properties of the GTVs as contoured by radiation oncologists are summarized in Table 1 and Figure 3. The volume of the GTVs varied from 0.1 cm^3^ to 103 cm^3^. The average distance between the center of mass of the GTVs based on the RIR and DIR was 1.8 mm with a standard deviation of 1.0 mm. The largest discrepancy observed was 4.5 mm in subject 5 who had a primary tumor located in the left tonsil. The average Dice similarity coefficient was 0.84 (95% confidence interval: 0.73-0.94). This excludes a subject who presented with a 0.1 cm^3^ primary node confined to the epiglottis, resulting in the RIR and DIR volumes not overlapping even though their spatial separation was measured to be only 1.6 mm. All GTVs received at least 95% of the prescribed dose to ≥ 99% of their volume. The difference in the mean dose to the GTVs drawn from the RIR and DIR was less than 0.5% in 17 out of 18 tumor sites, and was 2% for the secondary tumor of subject 5. The fraction of the volume of the GTVs receiving 100% of the prescribed dose (V100%) is presented in Table 2. The differences in V100% between RIR and DIR were at most 5%.

**Table 1:** Volumetric properties of the GTVs for the 10 head and neck cancer subjects

	**Primary gross tumor volume**				**Secondary gross tumor volume**			
**Subject**	**V**_**RIR**_	**V**_**DIR**_	**V**_**Overlap**_	**DSC**	**V**_**RIR**_	**V**_**DIR**_	**V**_**Overlap**_	**DSC**
	**(cm**^**3**^**)**	**(cm**^**3**^**)**	**(cm**^**3**^**)**		**(cm**^**3**^**)**	**(cm**^**3**^**)**	**(cm**^**3**^**)**	
1	20.1	15.2	14.3	0.81	12.1	8.1	8.0	0.79
2	52.3	48.0	41.3	0.82	1.4	1.4	1.2	0.86
3	25.3	23.3	22.0	0.91	9.9	8.3	8.6	0.90
4	7.8	8.7	7.4	0.90	3.7	3.8	3.3	0.88
5	18.6	18.9	14.2	0.76	20.4	23.4	16.8	0.77
6	102.9	91.5	87.3	0.90	6.3	5.5	5.4	0.92
7	0.1	0.1	0.0	0.00	na	na	na	na
8	4.4	5.6	3.9	0.78	na	na	na	na
9	26.7	19.1	18.2	0.79	1.1	1.0	0.8	0.76
10	10.4	10.7	9.0	0.85	7.2	7.1	5.8	0.81

The volumes corresponding to the GTVs drawn using the rigid and deformable image registration are labeled as V_RIR_ and V_DIR_, respectively. The acronym “na” means the GTV was not available.

**Table 2:** **Percentage of the volume of the GTVs receiving at least 100**% **of the prescribed dose**

	**Head and neck subjects**				**Lung subjects**			
**Subject**	**Primary node**		**Secondary node**		**Primary node**		**Secondary node**	
	**% V**_**RIR**_	**% V**_**DIR**_	**% V**_**RIR**_	**% V**_**DIR**_	**% V**_**RIR**_	**% V**_**DIR**_	**% V**_**RIR**_	**% V**_**DIR**_
1	78	80	82	87	94	95	na	na
2	64	69	93	93	98	99	na	na
3	61	60	99	98	100	100	100	100
4	100	100	99	99	100	100	na	na
5	83	78	98	96	100	100	91	91
6	69	72	100	100	97	97	100	100
7	100	100	na	na	100	100	na	na
8	99	100	na	na	89	94	na	na
9	100	100	76	76	100	100	83	84
10	100	100	100	100	67	67	31	31

The convention described in Table 1 is used.

### Lung cancer analysis

Ten lung cancer patients were retained for this study. The properties of the GTVs are presented in Table 3. Five subjects had secondary tumor volumes in the mediastinum or hilar region that were identified using PET images and contoured. The size of the GTVs varied from about 3 cm^3^ to over 350 cm^3^. The spatial difference between the center of mass of the GTV from the RIR and DIR was found to be consistently small for all patients, on average 0.6 mm with a standard deviation of 0.6 mm. These numbers increased to 0.7 mm when lymphatic secondary nodes were excluded. For all 10 patients with multiple nodes contoured, the exact same displacements along the longitudinal axis were observed for the GTVs, whereas displacements in the transverse plane varied (Figure 3). The average Dice similarity coefficient was 0.93 (95% confidence interval: 0.80-1.00), and 0.90 when excluding lymphatic nodes. All GTVs received at least 95% of the prescribed dose to ≥ 99% of their volume except for the secondary GTV of patient 10 where a significantly lower dose was delivered to the mediastinum to spare the heart and lungs.

**Table 3:** Volumetric properties of the GTVs for the 10 lung cancer subjects

	**Primary gross tumor volume**				**Secondary gross tumor volume**			
**Subject**	**V**_**RIR**_	**V**_**DIR**_	**V**_**Overlap**_	**DSC**	**V**_**RIR**_	**V**_**DIR**_	**V**_**Overlap**_	**DSC**
	**(cm**^**3**^**)**	**(cm**^**3**^**)**	**(cm**^**3**^**)**		**(cm**^**3**^**)**	**(cm**^**3**^**)**	**(cm**^**3**^**)**	
1	39.3	37.2	34.3	0.90	na	na	na	na
2	71.4	77.2	65.5	0.88	na	na	na	na
3	14.4	14.5	13.0	0.90	2.7	2.9	2.7	0.96
4	13.8	14.5	12.6	0.89	na	na	na	na
5	11.4	11.0	10.5	0.94	6.8	6.8	6.8	1.00
6	38.0	36.2	28.1	0.76	27.0	27.0	27.0	1.00
7	26.4	26.4	24.0	0.91	na	na	na	na
8	356.8	356.5	330.8	0.93	na	na	na	na
9	61.1	59.8	55.4	0.92	12.6	12.6	12.6	1.00
10	63.3	62.7	60.9	0.97	21.0	21.0	21.0	1.00

The convention described in Table 1 is used.

The difference in the average radiation dose received by the GTVs drawn from the RIR and DIR was less than 1% for all subjects. The differences in V100% between RIR and DIR were typically small and at most 5% as shown in Table 2.

## Discussion

Many studies investigating the performance and utility of DIR have been conducted. Schwartz et al. [10] performed DIR between planning CT and additional CT images acquired during the course of radiation for 22 head and neck cancer patients for the purpose of evaluating various adaptive radiotherapy techniques. They demonstrated that an adaptive radiation therapy process is feasible when using DIR and that improved sparing of organs at risk could be achieved. Castadot et al. [11], Fallone et al. [12] and Zhong et al. [13] performed a variety of phantom measurements to evaluate a deformable registration package and arrived at a protocol for systematic evaluation of DIR. Senthi et al. [14] quantified differences in RIR and DIR for 10 re-irradiated lung cancer patients for whom initial planning CTs were registered with a subsequent planning CT used in a second treatment plan. They observed improvements in registering organs at risk when using DIR as opposed to RIR; however, they did not evaluate possible changes in patient dosimetry. Similarly, Ireland et al. [15] quantified differences in RIR and DIR for five head and neck cancer patients for whom PET/CT scans were registered against planning CT images. They observed that DIR provided a more accurate registration than RIR for a set of anatomic landmarks, but did not evaluate differences in patient dosimetry. Yin et al. [16] evaluated a variety of DIR packages for the purpose of accurately registering normal tissue function (SPECT) with the planning CT. Despite these publications, no studies to date have evaluated possible differences in overall gross tumor volume (GTV) delineation, and possible changes in dose to the GTV, when using RIR or DIR between PET/CT and planning CT images.

Among all subjects with multiple tumors, there were consistent longitudinal differences in the location of the GTVs contoured with RIR and DIR. The internal RIR within the DIR algorithm may be the cause for the consistent difference in longitudinal location. This observation also suggests that DIR was performed on a slice-by-slice basis, that is, no deformations were performed longitudinally.

Although the size of the GTVs differed by as much as 30% between RIR and DIR, their locations were the same to within 4.5 mm and the Dice similarity coefficients were high for 32 out of 33 tumor volumes, indicating a high level of compatibility. In the case of the subjects with lung cancer, the location and size of the mediastinal and hilar GTVs were observed to be identical for 4 out of 5 patients because the radiation oncologists elected to treat a volume not only limited to FDG-avid node(s), but also incorporating neighboring lymph nodes suspected to be involved. In these cases, the GTVs were drawn based on patient anatomy using the planning and diagnostic CT scans.

Clinical target volumes were created around the RIR-defined GTV using margins of 5-8 mm for head-and-neck and 7 mm for lung cancer patients. Additionally, 4 mm margins were added to form the planning target volumes (PTV). External beam planning was then performed using the RIR-defined PTV. Hence, the radiation dose delivered to the RIR and DIR drawn GTVs were very similar as the DIR-defined GTVs were contained within the RIR-defined PTV.

The sparing of healthy tissues and organs at risk was not investigated given the small changes observed in the position between RIR-defined and DIR-defined GTV. Potential gains were assumed to be minimal.

### Limitations

It is of note that when applying the DIR to the PET images the intensity of the voxels (Bq/ml) is not preserved. This was observed to impact the maximum standardized uptake value by less than 1%, and is assumed to be negligible in the determination of GTVs when compared to random systematic uncertainties during manual contouring which have been shown to be important [18].

Although the lung tumors were located in the upper region of the lung where sensitivity to breathing motion is reduced, gating techniques during the acquisition of the planning CT and PET/CT would have further reduced the sensitivity to respiratory motion. The wide time range between the acquisition of planning CT and PET/CT scans of 1-15 days may have been a significant factor in apparent tumor motion, particularly for patients with rapidly developing tumors. Finally, the delineation of hilar and mediastinal nodes by radiation oncologists for lung cancer patients was often based on the anatomy rather than metabolic data, which may have biased the results. As such, the data for the lung cancer patients were presented with and without these secondary nodes.

## Conclusion

Deformable image registration has become an important component of image-guided and adaptive radiation therapy protocols. Commercial software to perform DIR is now available at the BC Cancer Agency for registering PET/CT images to planning CT scans, but this study revealed minimal benefits. Unless there are significant anatomical differences between the PET/CT and planning CT, the value of deformable registration between PET/CT and planning CT images was shown to be marginal value when delineating gross tumor volumes.

## Abbreviations

CT: Computed tomograpy; PET: Positron Emission Tomography; FDG: Fluorodeoxyglucose; RIR: Rigid image registration; DIR: Deformable image regisgration; GTV: Gross tumor volume; IMRT: Intensity modulated radiotherapy; DSC: Dice similarity coefficient; SUV: Standardized Uptake Value; PTV: Primary target volume.

## Competing interests

The authors declare that they have no competing interests.

## Authors’ contributions

DF participated in the study design, performed the registration, collated the results, performed the analysis and drafted the manuscript. PSB participated in the study design, analysis of the results and helped draft the manuscript. TB, DP, ESW performed the GTV contouring and participated in the study design. All authors read and approved the final manuscript.
